# Exploring Latent Profiles of Problematic Social Media Use and Their Relationship with Distress, FoMO, and Self-Esteem: A Cross-Sectional Study in a Turkish Adult Population

**DOI:** 10.1007/s11126-026-10269-4

**Published:** 2026-03-20

**Authors:** Abdullah Mücahit Aslan, Hayri Koç

**Affiliations:** 1 Department of Guidance and Psychological Counseling, Karamanoğlu Mehmetbey University, Karaman, Türkiye; 2 Department of Guidance and Psychological Counseling, Necmettin Erbakan University, Konya, Türkiye

**Keywords:** Problematic social media use, Distress, FoMO, Self-Esteem

## Abstract

This study explored problematic social media use (PSMU) among a sample of 1,338 Turkish adults (67.1% female, 32.9% male) aged 17–68 years (M = 24.18, SD = 8.15), representing 65 provinces in Türkiye. Latent profile analysis (LPA) was conducted based on item-level responses to the Bergen Social Media Addiction Scale (BSMAS), and multinomial logistic regression analyses were performed to examine associations between profile membership and psychological distress (Depression Anxiety Stress Scales–21; DASS-21), fear of missing out (FoMO), and self-esteem (Rosenberg Self-Esteem Scale; RSE). A latent profile analysis identified four distinct profiles of PSMU: Minimal Users (32.81%), Habitual Users (22.65%), Engaged Users (28.33%), and Heavy Addicts (16.22%). The 4-class model outperformed alternative solutions based on fit indices and classification accuracy (entropy = 0.73). Minimal Users exhibited infrequent social media use with no signs of addiction. Habitual Users integrated social media into their daily lives and reported negative experiences when attempting to reduce usage. Engaged Users displayed high social media engagement with controlled addictive tendencies, while Heavy Addicts demonstrated severe addiction with substantial life disruptions. Logistic regression analyses indicated that age was associated with a higher likelihood of membership in the Minimal User profile, whereas higher levels of depression, stress, and FoMO were associated with membership in more problematic profiles. Higher self-esteem was associated with a greater likelihood of belonging to the Minimal User profile compared to more engaged profiles. These findings underscore the heterogeneity of PSMU and highlight the psychological and demographic variables associated with profile membership. The study’s findings suggest that young adults with high levels of psychological distress, fear of missing out, and low self-esteem could be a priority group in future preventive and intervention efforts targeting problematic social media use.

## Introduction

Humans are inclined to adopt tools that facilitate daily functioning, and social media has become one of the most prominent digital tools of contemporary life. In the present study, social media refers to digital platforms and applications that enable users to create, share, and interact with user-generated content and social networks in real time, including social networking sites, media-sharing platforms, and messaging-based social applications [1, 2]. By enabling rapid access to information, communication, and large-scale content sharing, social media platforms serve important functional purposes in modern societies. However, the increasing centrality of social media in everyday life has also raised concerns regarding the potential shift from functional use to problematic patterns of engagement.

Recent global reports illustrate the widespread use of social media worldwide. As of January 2024, approximately 5.04 billion individuals were active social media users, representing about 62.3% of the global population, with an annual increase of 266 million users [3]. In Türkiye, social media use is similarly prevalent, with 54.30 million users aged 18 and above, accounting for approximately 86% of the adult population [4]. These figures underscore the societal relevance of social media use both globally and nationally, providing important context for examining problematic patterns of engagement.

Social media platforms serve multiple functional purposes, including facilitating social connection, identity expression, information seeking, and stress regulation. Previous research has shown that social media use may contribute to the development of social capital by enabling users to maintain interpersonal relationships, access social support, and experience a sense of belonging [5, 6]. In addition, social media environments provide opportunities for identity exploration and self-presentation, particularly during emerging adulthood, when identity-related processes are salient [7]. On the other hand, the tendency of individuals to pass extended amounts of time on social media platforms brings about the development of addiction [8]. Excessive use of social media can affects individuals’ lives similar to those of substance addiction [9]. Additionally, overuse social media usage must cause issues in psychological and relational life and persist in a problematic manner [9]. Globally, around 5–10% of social media users fall into the addiction spectrum [10]. Studies have indicated that PSMU is significantly associated with issues such as sleep disorders [11], excessive self-comparison and biased self-image [12], somatic symptoms [13], dark triad traits [14], low physical activity [15], low subjective well-being [16], loneliness [17], narcissism [18].

PSMU is conceptually distinct from high but non-problematic engagement with social media. While frequent or intensive use may reflect occupational, academic, or social demands, PSMU is characterized by impaired control, preoccupation, and negative consequences in daily functioning [19, 20]. Prior research has emphasized that frequency of use alone is insufficient to distinguish problematic from non-problematic engagement, underscoring the importance of symptom-based conceptualizations ([21]; Starcevic & Billieux, 2017).

In this regard, Griffiths’ components model of addiction provides a widely accepted theoretical framework for conceptualizing behavioral addictions, including problematic social media use. According to this model, addictive behaviors are defined by six core components: salience, mood modification, tolerance, withdrawal, conflict, and relapse [8, 9]. This framework has been extensively applied in the study of technology-related behavioral addictions and has informed the development of validated measurement tools. Consistent with this perspective, the Bergen Social Media Addiction Scale (BSMAS) operationalizes PSMU by directly assessing these addiction components at the item level, rather than relying on usage frequency or duration alone [22].

A key focus of our research is the connection between PSMU and mental health. The concept of psychological distress, characterized by stress, depression, and anxiety is considered a general measure of individuals’ mental health [23]. It is considered that investigating the interactions between PSMU and psychological distress can provide important information about the relationship between the mental health of individuals and PSMU. When examining studies in the literature, PSMU has been found to have significant associations with high levels of depression [24–27], anxiety [28–30], and stress [31, 32]. Possible reasons for these results have been suggested to include constant exposure to the perception on social media that others are happier and live higher standards of life [33]. Another reason is the excessive time spent on social media, which not only reduces physical activities but also leads to the formation of dysfunctional cognitions, thereby contributing to psychological distress [34]. Furthermore, it has been observed that individuals experiencing psychological distress frequently utilize social media as a means of regulating their emotions [35]. For certain individuals, Use social media to emotion regulation may cause excessive use, driven by a deep-seated fear of missing out on updates and events.

FoMO describes the fear of missing out on enjoyable moments others might be experiencing, driving a constant need to keep track of their actions [36]. One of the reasons for addressing FoMO in our study is its association with concepts such as excessive internet use (Koca & Saatcı, 2022; [37]) and problematic smartphone use [38, 39], as well as its relationship with PSMU [40–42]. Studies suggest that FoMO plays a crucial role in predicting PSMU. [43–45]. In addition, experiencing high levels of FoMO can adversely impact individuals’ lives. Research indicates that FoMO is strongly associated with increased levels of anxiety and depression [39, 46], stress [47], negative emotions [48], and neuroticism [49].

Some researchers have explained the significant relationships between FoMO and PSMU through the need to engage with others [50]. PSMU accompanied by FoMO are associated with outcomes that may affect individuals’ levels of self-esteem. Self-esteem is the degree to which a person feels valuable and the state of having either a positive or negative judgment about themselves [51]. A person’s self-perception and evaluations are the key factors that determine self-esteem; if a person evaluates themselves negatively, they will have low self-esteem, while if they evaulates themselves in a positive way, it results in higher self-esteem. Rosenberg, [52]. Studies have demonstrated that low self-esteem is significantly linked to emotional issues like depression, stress, and anxiety. Kircaburun, [53], Orth, [54]. Building on these research findings, it can be concluded that high self-esteem has positive effects on mental health. Moreover, there is evidence in the literature that PSMU, which is the dependent variable in our research, is significantly related to low self-esteem [55–58]. Research investigating the connection between Facebook addiction and self-esteem found that individuals with Facebook addiction tend to have lower self-esteem. Błachnio et al., [59]. A study conducted on 23,532 Norwegian individuals also revealed that overuse of social media has an adverse relationship with participants’ self-esteem evaluations [18]. The results of these studies suggest that an important factor influencing self-esteem is the comparison people make between themselves and others on social media [55]. Such social comparisons have a weakening effect on self-esteem [60].

Demographic factors, particularly age and sex, have also been examined as correlates of problematic social media use, although findings remain mixed. Several studies have reported higher levels of problematic social media use among younger individuals, suggesting that developmental stage, identity exploration, and greater integration of social media into daily life may increase vulnerability to problematic patterns [2, 18, 61]. In contrast, other studies have found weaker or non-significant age effects, indicating that problematic use may persist across adulthood under certain psychological conditions [62]. With respect to sex, some research has suggested that females may report higher levels of social media engagement and related problematic use, potentially due to greater emphasis on social interaction and online self-presentation [22, 63]. However, other studies have reported minimal or inconsistent sex differences, underscoring the need to examine age and sex within broader psychological and contextual frameworks rather than as isolated predictors [20, 21]. These mixed findings highlight the importance of considering demographic variables alongside psychological factors when examining heterogeneity in PSMU.

Numerous variable-centered researches have explored the relationships between PSMU and factors such as stress, depression, anxiety, self-esteem, and FoMO. In variable-centered studies, all individuals in the sample are treated as members of a homogeneous, unique group, and the differences within the sample are not sufficiently identified, which can cause discrepancies in the results across studies. Lee and Yoo, [64]. In the context of behavioral addictions such as PSMU, this limitation is particularly problematic, as individuals may reach similar levels of overall symptom severity through qualitatively different psychological pathways. Consequently, variable-centered findings may conflate distinct subgroups, weaken theoretical interpretations, and contribute to inconsistent associations across studies, especially when different psychological correlates are examined. In contrast, person-centered approaches aim to identify previously unrecognized differences within a study population [65]. Latent Profile Analysis (LPA) is a person-centered analytical method that examines heterogeneity within a population. Person-centered approaches aim to categorize individuals into distinct groups based on their responses to multiple variables [66]. As a result, person-centered approaches allow researchers to obtain a more detailed understanding of the traits and characteristics of different groups of individuals. Thus, LPA was selected to explore the presence of distinct subgroups of individuals based on PSMU within the sample. The findings of this study are expected to provide a deeper understanding of how individuals’ PSMU profiles are associated with external factors such as anxiety, depression, stress, self-esteem, and FoMO.

The present study aims to identify distinct latent profiles of problematic social media use (PSMU) using a person-centered approach and to examine how psychological distress (depression, anxiety, and stress), fear of missing out (FoMO), and self-esteem are associated with membership in these profiles. By doing so, the study seeks to clarify the psychological correlates of heterogeneous PSMU patterns in a Turkish adult sample.

## Research Gap and Rationale

Although an increasing number of studies have adopted person-centered approaches to examine problematic social media use, several important gaps remain in the literature. First, existing latent profile and latent class analyses have predominantly focused on adolescents or university student samples, thereby limiting the generalizability of findings to broader adult populations (e.g., [61, 63]). Person-centered investigations examining heterogeneous patterns of problematic social media use across a wide adult age range, particularly within non-Western cultural contexts such as Türkiye, remain scarce.

Second, prior person-centered research has often examined a restricted set of psychological correlates, frequently focusing on single variables (e.g., anxiety or depression) rather than integrating multiple theoretically relevant constructs within a unified analytical framework [21, 39]. Consequently, limited attention has been given to how psychological distress, FoMO, and self-esteem jointly relate to distinct patterns of problematic social media use.

Addressing these gaps, the present study applies a person-centered latent profile analysis approach to identify distinct profiles of problematic social media use in a large and demographically diverse sample of Turkish adults. By simultaneously examining psychological distress, FoMO, and self-esteem as correlates of profile membership, the study aims to provide a more comprehensive, integrative, and culturally contextualized understanding of heterogeneity in problematic social media use.

### Aims

The study is focused on two main objectives. The first is to identify how individuals differ in terms of PSMU or to determine which latent profiles they possess. The second objective is to examine the relationships between latent profile memberships and the variables of psychological distress, self-esteem and FoMO. In accordance with these goals, the importance of this study is to determine the latent profiles related to PSMU and to investigate the connections between the variables specified with the latent PSMU profiles with a person-centered approach. Thus, to determine the factors associated with PSMU profiles and to benefit from them in intervention processes.

### Theory

Linking PSMU to individual, behavioral, emotional, and psychological variables can be specifically examined within the context of the Interaction of Person-Affect-Cognition-Execution (I-PACE) model, Compensatory Internet Use Theory (CIUT) and Self-Determination Theory (SDT). Among these, SDT, developed by Deci and Ryan [67], emphasizes that the sources of human behavioral motivation are the needs for relatedness, autonomy, and competence. Within the framework of SDT, social media can be considered an important tool for fulfilling these three basic needs [42, 68, 69]. Individuals can satisfy their needs for social connection and belonging by interacting with others via social media platforms. Additionally, the likes and comments received on social media can meet individuals’ competence needs. Being able to share the content they want on social media, make the comments they want, and connect with the people they want or end their existing connections can provide them with important opportunities to meet their need for autonomy. It is well known that these needs are universal and that fulfilling them plays important roles in individuals’ health and well-being [70]. However, certain individuals can link to social media in a problematic way to fulfill these needs within the SDT framework [71, 72]. If social media cannot efficient meet these needs, or if individuals feel inadequate when comparing themselves to others’ idealized social media profiles, they may experience disappointment, stress, and depression [33]. In terms of relatedness needs, frequently checking social media to keep track of friends compulsively can result in the development of FOMO and anxiety [36]. Furthermore, in relation to SDT, the satisfaction of competence needs through likes and positive comments can also support the increase of low self-esteem [73].

Another important foundation for our research is the I-PACE model [74]. The model explains addictive behaviors by examining the interaction of individual variables such as personality traits (person), emotional processes (affect), cognitive processes (cognition), and executive functions (execution). When examining PSMU within the I-PACE framework, self-esteem in our study can be interpreted regarding personality traits. Individuals may engage with social media more frequently to support their self-esteem through likes and positive comments within this model [75]. Within the context of emotional processes, people who are struggling with depression, anxiety, and stress may use social media to regulate their moods, manage emotional states, or escape from these emotional states [76]. In terms of cognitive processes, it is known that FOMO, accompanied by dysfunctional beliefs and compulsions, can drive individuals to be frequently online on social media to avoid missing out on developments. As a result of all these processes, in terms of executive functions in the model, individuals’ social media use can become unmanageable, impulsive, and reach problematic levels. The fundamental view of the CIUT, put forward by Kardefelt-Winther [77], is that the web is used to compensate for the absence of reality. In this context, people may go online or to social media platforms accessed via the Internet as a way to cope with psychological distress and regulate their emotional states [76]. Individuals use the internet or internet-based platforms in a problematic way when face-to-face communication is not possible to alleviate their psychological distress ([78]; O’Farrell et al., 2020; [72]). Based on this, within the CIUT framework, emotional, cognitive, and behavioral elements like distress, FOMO, and low self-esteem can be seen as precursors to PSMU.

When the models are examined in general, SDT suggests that unmet needs serve as the motivational sources for behaviors related to PSMU, while I-PACE explains the formation of PSMU through the interactions of the individual, emotions, cognition, and executive processes. CIUT, on the other hand, suggests that psychological problems and coping behaviors serve as factors associated with PSMU. These models not only explain the formation of PSMU but also provide important insights for interventions, such as developing intrinsic motivation, addressing needs in healthy ways, and improving self-regulation skills to cope with it.

### Studies on Profiles of PSMU

Previous research on PSMU has primarily relied on variable-centered approaches, classifying individuals based on their standard deviation and mean scores. For instance, in a study by Andreassen et al., [79], individuals were categorized as problematic and non-problematic users, while Müller et al., [80] identified three groups: non-addicted, problematic, and addicted. These studies, being variable-centered, fail to account for the heterogeneity within the study group, thus not providing entirely accurate profile information [81]. The LPA method, using an individual-centered approach, maximizes inter-group differences and minimizes intra-group differences, thus defining the latent profiles more accurately based on the observed data [25]. Studies using LPA reveal that the number of latent profiles identified for PSMU varies across studies. For instance, Cui et al., [78] examined the LPA of Chinese undergraduates concerning PSMU and identified four profiles: high-risk group, enjoyment-focused medium-risk group, compulsion-focused low-risk group and medium-risk group. The study also investigated the relationships between these four latent profile memberships and stress and depression. Significant differences were found in the stress scale between the low-risk groups and high-risk, and in the depression scale between the enjoyment-focused medium-risk and compulsion-focused medium-risk groups. Similarly, Cheng et al., [25] performed a research on British and American samples to determine latent profiles and their associations with four main classification schemas. They identified three latent profiles in both cultures: low-risk, at-risk, and high-risk. In the research conducted by Keum et al., [82], social media usage profiles of young adults were determined according to their social media usage frequency and purposes, and the relationship between these profiles and positive and negative impacts they have on psychosocial well-being. was examined. Three latent profiles were determined: passive users (25.3%), active users (32.4%), and average users (42.4%). The study concluded that while active users experienced enhanced psychosocial well-being, they also encountered more negative consequences. In contrast, passive users gained less benefit from social media but faced fewer issues related to problematic use and stress.

An examination of the existing literature reveals that a steadily expanding body of research has utilized person-centered techniques, especially LPA and latent class analysis (LCA), to investigate heterogeneity in social media use. However, existing person-centered studies vary substantially in their conceptualization of social media use, measurement strategies, and sample characteristics. For example, some studies have focused on general or intensive social media engagement based on usage frequency or time spent online rather than symptom-based problematic use (e.g., Marino et al., [63]; [25]). In contrast, other studies have examined problematic social media use but have relied on different operationalizations or platform-specific indicators, limiting comparability across findings [61, 83]. Moreover, the majority of existing LPA studies on social media use have been conducted with adolescents or university student samples (e.g., Bányai et al., [61], Cui, [78], Marino, [63], and predominantly within Western cultural contexts. This focus restricts the generalizability of person-centered findings to broader adult populations and non-Western settings. Importantly, not all prior person-centered studies have employed addiction-based measures grounded in established theoretical frameworks, such as Griffiths’ components model of addiction. Many studies have relied on behavioral indicators of engagement rather than symptom-based assessments capturing impaired control, salience, or functional impairment [20, 21]. In addition, previous LPA research has typically examined psychological correlates—such as distress, fear of missing out (FoMO), or self-esteem—in isolation, rather than integrating multiple vulnerability factors within a single analytical framework [39, 63].

Building on these limitations, the present study applies latent profile analysis to item-level responses from the Bergen Social Media Addiction Scale (BSMAS), a measure explicitly grounded in Griffiths’ components model, to identify distinct profiles of problematic social media use in a large and demographically diverse Turkish adult sample. By simultaneously examining psychological distress, FoMO, and self-esteem as correlates of profile membership, this study extends prior person-centered research by providing a more integrative and theoretically grounded understanding of heterogeneity in PSMU.

### Hypotheses

- *H1. LPA conducted based on the responses to the items of the Bergen Social Media Addiction Scale (BSMAS) should reveal latent profiles in the range of three to five.*

Based on prior latent profile and latent class studies on social media use and related problematic digital behaviors, which have consistently identified between three and five distinct subgroups across different populations and measurement approaches (e.g., Bányai et al., [61]; [25, 78]), the present study hypothesized that a similar range of latent PSMU profiles would emerge. When previous studies are examined, it can be observed that Cui et al., [78] carried out a study involving Chinese university students and identified four distinct classes. Cheng et al., [25] three classes identified in both American and British samples; Keum et al.,; [82] found three classes in a study with American young adults; Bányai et al.,; [61] identified three classes in Hungarian adolescents; and Ilakkuvan et al., and [83] found five classes in American young adults aged 18–24. Based on these studies, it is anticipated that our research will also yield either three or five profiles.

- *H2. Stress*,* anxiety*,* depression*,* and FOMO severities are expected to be positively associated with the most severe latent profile membership based on PSMU.*

Studies have revealed positive and significant relationships between PSMU and external variables like depression [24–26], anxiety [28, 29], stress [31, 32], FOMO [40–42]. When individuals are unable to fulfill their needs for attachment, autonomy, and competence through face-to-face communication, they experience psychological distress. To reduce this distress and fulfill unmet needs, they develop PSMU, which is consistent with SDT (O’Farrell et al., 2020; [72]) and CIUT [76, 78]. Additionally, the I-PACE model points to emotional variables like stress, anxiety, and depression, as well as cognitive and behavioral factors such as FOMO, as key correlates of PSMU [76].

- *H3. Self-esteem is expected to be negatively associated with the most severe latent profile membership based on PSMU.*

Studies have revealed strong negative relationships between PSMU and high self-esteem [55, 57, 58]. It is considered consistent with the I-PACE model that self-esteem can be evaluated as a personal trait within the model, and that low self-esteem might significantly are related to the development of PSMU through interaction with other emotional, cognitive, and executive processes. Seabrook et al., [75], Soraci, [84]. Additionally, people with low self-esteem may utilize social media as a means to satisfy their need for a sense of competence (such as positive comments, likes etc.), and with the reinforcement of this cycle, the development of PSMU can be seen as consistent with SDT (Sommantico et al., 2023). Additionally, the problematic use of online-based platforms by people to regulate their self-esteem aligns with CIUT [85].

## Method

### Participant and Procedure

In the current study, a cross-sectional methodology was used and data collected by a internet-based survey in Türkiye between November 01 and November 30, 2024. Participants were recruited using a convenience sampling approach, where researchers shared the survey link through widely used social media networks such as WhatsApp, X, and Telegram. Inclusion criteria for the study were: (1) being at least 17 years of age, (2) residing in one of the provinces of Türkiye, and (3) being an active user of at least one social media platform. Prior to data collection, participants were presented with an electronic informed consent form outlining the study’s purpose, duration, and data privacy protocols. Consent was documented via a mandatory digital tick-box (‘I agree’); participants who did not provide consent were automatically exited from the system. To ensure data quality, the survey was closed automatically after a 30-day response period. Participation was strictly voluntary, and no financial compensation or other incentives were provided to the participants for their involvement in the study. The sample includes 1338 Turkish individuals (898 [67.1%] female and 440 [32.9%] male) aged 17–68 years (M = 24.18, SD = 8.15) from 65 of the 81 cities in Turkey. The educational status of the sample participants was as follows: 1.1% were elementary school graduates (*n* = 15), 1.5% were middle school graduates (*n* = 20), 13.2% were high school graduates (*n* = 176), 14.0% were associate degree graduates (*n* = 187), 66.4% were bachelor degree graduates (*n* = 888), and the rest (*n* = 52) were master’s degree and above (3.9%).The majority of the participants provided data regarding their socioeconomic status as medium (*n* = 1166, 87.1%). Other participants reported low (*n* = 111, 8.3%) and high (*n* = 61, 4.6%) socio-economic status. The most frequently utilized social media platform of the participants were: Instagram (*n* = 722, 54%), WhatsApp (*n* = 340, 25.4%), YouTube (*n* = 122, 9.1%), X (*n* = 107, 8%), Facebook (*n* = 23, 1.7%). In addition, a few participants (1.8%) stated that they most frequently use applications such as TikTok, Telegram, Spotify, LinkedIn, Discord, Pinterest. 23 participants (1.7%) spend less than 30 min a day on social media. 127 participants (9.5%) spend between 30 min and one hour a day on social media. 301 participants (22.5%) spend 1–2 h a day on social media. 352 participants (26.3%) spend 3–4 h a day, 278 participants (20.8%) spend 4–5 h, 11.4% spend 4–5 h, and 7.8% spend more than 5 h.

### Instruments

#### **Bergen Social Media Addiction Scale (BSMAS)**

This instrument is a 6-item self-assessment instrument developed by Andreassen et al., [22] to measure the level of problematic social media use. Participants evaluate their experiences in the past year using a five-point Likert scale ranging from 1 (very rarely) to 5 (very often). Although the BSMAS is primarily a screening tool, various cut-off scores have been proposed to identify problematic use. For instance, Luo et al., [10] recommended a cut-off score of 24 for determining the risk of social media addiction. In the current study, we employed Latent Profile Analysis to identify subgroups based on response patterns rather than relying on a single clinical cut-off, which allows for a more nuanced understanding of the heterogeneity within the population. The scale measures the fundamental aspects of addiction and has exhibited strong internal consistency in a Turkish sample [86]. In this study, the BSMAS exhibited sufficient reliability, with a Cronbach’s alpha of 0.77.

#### **Depression Stress and Anxiety Scale (DASS21)**

This instrument is a self-assessment instrument consisting of 21 items [87, 88]. The DASS21 consists of three subscales, with each subscale containing seven items: depression, stress, and anxiety. All items are evaluated using a four-point scale, with ratings ranging from 0 (indicating that the item was not applicable to the respondent) to 3 (indicating that the item was highly applicable to the respondent). Participants are instructed to respond to the scale based on their experiences over the previous seven-day period. The DASS-21 provides three subscale scores, each ranging from 0 to 21. In the current study, these subscale scores were treated as continuous covariates in the logistic regression model to predict latent profile membership. No clinical severity cut-offs were applied to categorize the participants; instead, raw subscale scores were used to maintain data granularity. The DASS21 has demonstrated robust internal consistency in Turkish samples [89]. In the current research, the scale demonstrated satisfactory internal consistency, with Cronbach’s alpha coefficients of 0.86 for depression, 0.84 for anxiety, and 0.82 for stress.

#### **Fear of Missing out Scale (FoMOS)**

This instrument is a ten-item self-assessment scale [36] utilized to evaluate the extent of fear of missing out. All elements are rated on a 5-point Likert scale, ranging from 1 (showing that the item is not at all true) to 5 (indicating that the item is absolutely true). Total scores for the FoMOS range from 10 to 50, with higher scores reflecting higher levels of the fear of missing out. The total score was used as a continuous variable in all statistical analyses, and no specific cut-offs were utilized to classify participants. The internal reliability of FoMOS has been demonstrated to be robust in Turkish samples [90]. In the present study, the scale showed satisfactory internal consistency, with a Cronbach’s alpha coefficient of 0.83.

#### **Rosenberg Self-Esteem Scale (RSE)**

This instrument is a 10-item self-report scale [52] utilized to assess the self-esteem. All items are rated on a 4-point Likert scale, ranging from 1 (indicating that the item is —strongly disagree) to 4 (indicating that the item is strongly agree). A specimen of an item is, “I feel that I have a number of good qualities.” The total score for the RSE ranges from 10 to 40, where higher scores represent higher levels of self-esteem. For this study, total scores were analyzed as a continuous covariate. Following the original scale guidelines, no clinical cut-offs were applied to categorize levels of self-esteem. The internal reliability of RSE scale has been demonstrated to be robust in Turkish samples [91]. The scale demonstrated acceptable internal consistency in the current study, with a Cronbach’s alpha coefficient of 0.86.

### Data Analysis

Data analyses were conducted using Mplus version 8.3 for Latent Profile Analysis (LPA) and SPSS version 25.0 for descriptive statistics, correlations, and multicollinearity diagnostics. The final sample for analysis consisted of 1,338 participants. As the data were collected via a web-based survey with mandatory response settings for all scale items, there were no missing data among the indicators used for the Latent Profile Analysis or the covariates used in the multinomial logistic regression models. Therefore, no data imputation or deletion methods were required. The six individual items of the Bergen Social Media Addiction Scale (BSMAS) were used as continuous indicators for the latent profiles. To determine the optimal number of profiles, we compared models with one to five classes using several fit indices: the Bayesian Information Criterion (BIC), sample-size adjusted BIC (aBIC), entropy, the Lo-Mendell-Rubin Likelihood Ratio Test (LMR), and the Adjusted LMR (aLMR). Lower values for BIC and aBIC indicate a better-fitting model, while entropy values closer to 1.00 suggest higher classification accuracy. Significant p-values for the LMR and aLMR tests indicate that the k-class model provides a significantly better fit than the k-1 class model. Final model selection was based on a combination of these statistical indices, model parsimony, and the theoretical interpretability of the identified profiles. Regarding the inclusion of covariates (age, sex, depression, anxiety, stress, FoMO, and self-esteem), a three-step approach was employed. In this procedure, the latent profiles were first identified based on the BSMAS items, and subsequently, the covariates were added to the model using multinomial logistic regression to predict profile membership. Given the multiple predictors and profile comparisons performed within the multinomial logistic regression, we acknowledge the potential risk of Type I error inflation. To mitigate this without utilizing overly conservative formal corrections that might obscure meaningful patterns in a large-scale sample, we prioritized the interpretation of results that showed consistency across different profile comparisons and relied on the magnitude of odds ratios and the precision of 95% confidence intervals rather than p-values alone.

## Results

### Descriptive Statistics and Correlations

Correlation coefficients and descriptive statistics for the variables investigated in this study are shown in Table 1. The preliminary assessment indicated that the kurtosis and skewness coefficients for the variables exhibited values between − 0.515 and 0.642. These ratios were below the established threshold of 2, indicating that the data had acceptable distributional characteristics, which are used to analyze the following according to George and Mallery (2021). The results of the correlation analysis indicated a statistically significant positive relationship between PSMU, distress, and FoMO variables. Also, self-esteem demonstrated a significant negative correlation with all other variables (see Table 2).

**Table 1:** Descriptive statistics

	Min	Max	M	SD	Skewness	Kurtosis
PSMU	6.00	30.00	16.676	5.241	0.075	−0.521
Depression	0.00	21.00	7.521	5.364	0.451	−0.642
Anxiety	0.00	21.00	6.685	5.010	0.515	−0.522
Stress	0.00	21.00	8.120	4.997	0.247	−0.602
FoMO	10.00	50.00	22.605	7.667	0.468	−0.214
Self-esteem	10.00	40.00	30.814	6.315	−0.483	−0.411

**Table 2:** Correlation among variables

			[2]	[3]	[4]	[5]	[6]
PSMU	[1]		0.424**	0.389**	0.433**	0.447**	−0.319**
Depression	[2]		-	0.708**	0.771**	0.483**	−0.626**
Anxiety	[3]			-	0.777**	0.454**	−0.467**
Stress	[4]		-		-	0.502**	−0.472**
FoMO	[5]			-	-	-	−0.341**
Self-esteem	[6]		-	-	-	-	-

**p<.01

To address potential multicollinearity concerns due to high correlations among variables, multicollinearity diagnostics were performed. Variance Inflation Factor (VIF) values for variables were found to be well below the threshold of 5 (ranging between 1.386 and 3.442), and tolerance values were above 0.10, indicating that multicollinearity did not pose a significant threat to the validity of the logistic regression models.

### LPA Model Selection

As shown in Table 3, the 4-class model demonstrated superiority over the other models, exhibiting lower BIC and aBIC values and significant LMR and aLMR values. Furthermore, the 4-class solution yielded a higher entropy value compared to the 3-class solution, suggesting enhanced classification accuracy. Although BIC and aBIC values continued to decrease for the 5-profile model, the LMR and aLMR tests were not statistically significant (*p* >.05), suggesting that a 5-profile solution did not provide a significantly better fit than the 4-profile model. Therefore, the 4-profile model was retained for its statistical parsimony and theoretical interpretability.

**Table 3:** Model comparisons for BSMAS item latent class analysis

Model	BIC	aBIC	Entropy	LMR	*p*	aLMR	*p*
1	26720.272	26682.154	NA	NA	NA	NA	NA
2	25308.732	25248.377	0.749	1461.933	< 0.001	1433.487	< 0.001
3	25081.174	24998.583	0.726	277.950	< 0.001	272.542	< 0.001
4	24964.227	24859.401	0.733	167.339	< 0.001	164.083	< 0.001
5	24903.028	24775.966	0.772	111.591	0.076	109.420	0.080

### Profile Descriptions

**Fig. 1:**
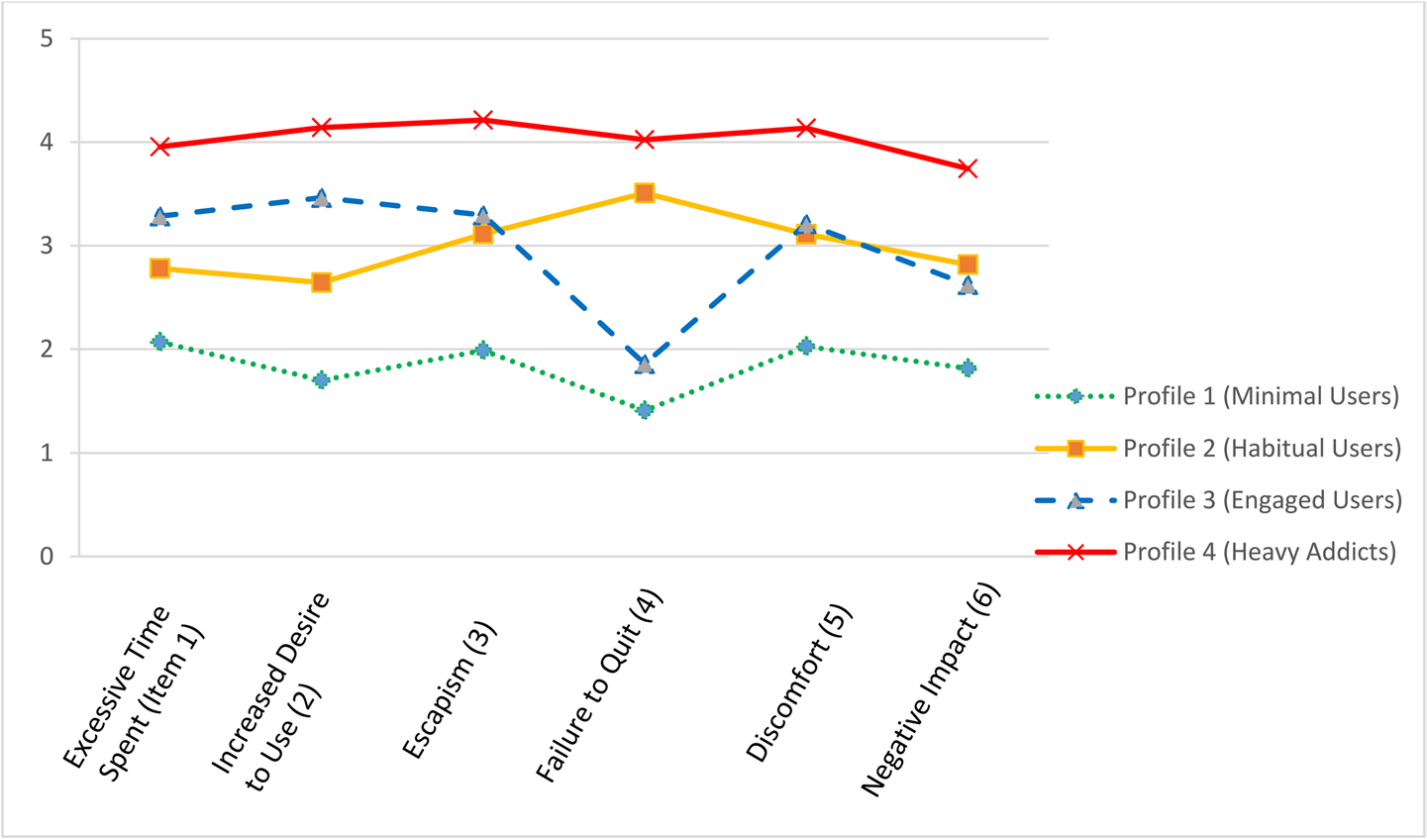
The Four-Profile LPA solution based on the six bsmas item using mean item scores

As demonstrated in Table 4, the four-profile model accurately classified 93% of Profile 1 participants, 80% of Profile 2, 80% of Profile 3, and 86% of Profile 4 participants. The 4-class solution was characterized as minimal users (32.81%), habitual users (22.65%), engaged users (28.33%), and heavy addicts (16.22%). Minimal Users are users who utilize social media infrequently or do not exhibit signs of addiction. Habitual Users: Participants who have incorporated social media into their daily routines, and whose experiences with ceasing to use social media have been predominantly negative. Engaged Users are users who extensively use social media and demonstrate addictive tendencies, yet their use remains under control. Heavy Addicts are users whose social media use severely impacts their lives and who show signs of intense addiction. Figure 1 shows the standardized mean BSMAS item scores for the four-profile model. Beyond the BSMAS item scores, the profiles also exhibited distinct sociodemographic compositions (see Table 4). Regarding sex, the Heavy Addicts profile was predominantly female (78.8%), whereas the Minimal Users profile had the highest representation of males (40.3%) compared to other groups. Educational background also varied across profiles; for instance, while 80.2% of Habitual Users held a bachelor’s degree, this rate dropped to 45.6% in the Heavy Addicts profile, where the majority (54.4%) belonged to the ‘Other’ education category (high school or associate degree). Conversely, socioeconomic status (SES) remained largely consistent across all groups, with ‘Medium’ SES being the most frequent category (ranging from 83.4% to 88.2%) in every profile.

**Table 4:** Four-Class LPA model participant distribution, Membership probability and sociodemographic characteristics

					Latent Profile Membership Probability				
Profile	Person		Percentage	Minimal Users		Habitual Users	Engaged Users	Heavy Addicts	
Profile 1	439		32.80%	0.927		0.021	0.053	0.000	
Profile 2	303		22.65%	0.052		0.800	0.086	0.062	
Profile 3	379		28.33%	0.069		0.103	0.798	0.030	
Profile 4	217		16.22%	0.000		0.068	0.069	0.863	
**Sex**									
Female	898		67.1%	262 (59.7%)		196 (64.7%)	269 (71.0%)	171 (78.8%)	
Male	440		32.9%	177 (40.3%)		107 (35.3%)	110 (29.0%)	46 (21.2%)	
**Education**									
Bachelor’s Degree	888		66.4%	284 (64.7%)		243 (80.2%)	262 (69.1%)	99 (45.6%)	
Other (High school/Assoc.)	450		33.6%	155 (35.3%)		60 (19.8%)	117 (30.9%)	118 (54.4%)	
**SES**									
Low	111		8.3%	31 (7.1%)		29 (9.6%)	30 (7.9%)	21 (9.7%)	
Medium	1166		87.1%	387 (88.2%)		264 (87.1%)	334 (88.1%)	181 (83.4%)	
High	61		4.6%	21 (4.8%)		10 (3.3%)	15 (4.0%)	15 (6.9%)	

### Regression Analyses

Table 5 presents the logistic regression coefficients and odds ratios (ORs) for the associations between the covariates and BSMAS latent profile membership, with the minimal users used as the reference profile. As age increases, the probability of falling into the minimal users also increases. A lack of statistically significant differences was observed between the profiles with regard to sex. Although the descriptive distribution in Table 4 indicates some proportional differences across groups—most notably a higher concentration of female participants in the Heavy Addicts profile (78.8%)—the multinomial logistic regression results revealed that sex was not a statistically significant predictor of profile membership. It should be noted that while education and socioeconomic status were examined descriptively, they were not included as predictors in the regression model. This suggests that the observed descriptive variations in sex do not carry unique predictive power once psychological factors such as FoMO, distress, and self-esteem are included in the model. The comparison between minimal users and habitual users indicates that greater levels of depression and FoMO are associated with a greater likelihood of belonging to habitual users. Similarly, a comparison between engaged users and minimal users demonstrates that a one-point increase on the FoMO scale corresponds to a 5.8% greater likelihood of being categorized into engaged users. Furthermore, elevated self-esteem is linked to a greater likelihood of membership in minimal users compared to engaged users. Lastly, the comparison between heavy addicts and minimal users reveals that higher scores on anxiety, stress, and FoMO scales are associated with an increased probability of falling into heavy addicts, whereas higher self-esteem is associated with a greater likelihood of membership in minimal users compared to heavy addicts. Furthermore, alternative parameterizations were analyzed by modifying the reference profile. The only parameter that differs from engaged users when habitual users is used as the reference is FoMO (B = −0.031, SE = 0.012, z = −2.643, *p* =.008, OR = 0.969, 95% CI [0.947, 0.993]). An increase in FoMO scores is associated with an elevated probability of entering the habitual users profile. When the engaged users is taken as a reference, an increase in FoMO (B = 0.051, SE = 0.014, z = 3.672, *p* <.001, OR = 1.052, 95% CI [1.024, 1.082]) and stress scores (B = 0.081, SE = 0.033, z = 2.455, *p* =.014, OR = 1.084, 95% CI [1.016, 1.157]) is associated with an elevated probability of entering the heavy addicts relative to the engaged users.

**Table 5:** Membership in the BSMAS latent profile (four-class model) and relationships with covariates

Covariate	B	SE of B	z	*p*	Odds Ratio	95% CI for OR
Habitual Users						
Sex	−0.226	0.164	−1.377	0.169	0.797	0.578–1.100.578.100
Age	−0.024	0.009	−2.602	0.009	0.976	0.959–0.994
Depression	0.057	0.027	2.091	0.037	1.058	1.004–1.116
Anxiety	−0.016	0.027	−0.595	0.552	0.984	0.933–1.038
Stress	0.038	0.028	1.385	0.166	1.039	0.983–1.097
FoMO	0.087	0.013	6.756	< 0.001	1.091	1.063–1.119
Self-esteem	−0.025	0.018	−1.388	0.165	0.975	0.942–1.010
Engaged Users						
Sex	0.039	0.167	0.233	0.816	1.040	0.750–1.442
Age	−0.024	0.010	−2.459	0.014	0.976	0.957–0.996
Depression	0.029	0.028	1.065	0.287	1.030	0.974–1.087
Anxiety	0.038	0.027	1.392	0.164	1.039	0.985–1.095
Stress	0.015	0.029	0.514	0.607	1.015	0.959–1.074
FoMO	0.056	0.013	4.296	< 0.001	1.058	1.031–1.085
Self-esteem	−0.044	0.018	−2.485	0.013	0.957	0.924–0.991
Heavy Addicts						
Sex	−0.453	0.215	−2.102	0.056	0.636	0.417–0.969
Age	−0.025	0.015	−1.715	0.046	0.975	0.947–1.004
Depression	−0.009	0.031	−0.278	0.781	0.991	0.933–1.053
Anxiety	0.087	0.032	2.678	0.007	1.091	1.025–1.162
Stress	0.096	0.034	2.837	0.005	1.101	1.030–1.177
FoMO	0.107	0.016	6.894	< 0.001	1.113	1.079–1.148
Self-esteem	−0.052	0.022	−2.383	0.017	0.950	0.909–0.991

Minimal Users is the reference for comparison

The four latent profiles were numerically distinguished by their mean scores on the BSMAS items and psychological covariates. Descriptive statistics for each profile are presented in Table 6. The results revealed a clear severity continuum across the profiles. The Minimal Users profile reported the lowest scores across all problematic indicators and psychological distress scales, alongside the highest levels of self-esteem (M = 33.37, SD = 6.62). Conversely, the Heavy Addicts profile exhibited the highest mean scores for PSMU (M = 17.61, SD = 4.80), depression (M = 8.44, SD = 5.28), anxiety (M = 7.62, SD = 5.06), and stress (M = 8.72, SD = 4.82). This group also reported the highest levels of FoMO (M = 24.32, SD = 7.49) and the lowest levels of self-esteem (M = 29.79, SD = 5.34).The Habitual Users and Engaged Users represented intermediate groups. While their PSMU scores were relatively similar (16.83 and 17.56, respectively), the Engaged Users profile was characterized by higher levels of anxiety (M = 7.20) and stress (M = 8.67) compared to the Habitual Users. These findings provide the numerical basis for the qualitative labeling of the profiles, where the Heavy Addicts profile represents the most psychologically vulnerable group.

**Table 6:** Means and standard deviations of psychological covariates across the four latent profiles of PSMU

Variable	Minimal Users (M\SD)	Habitual Users (M\SD)	Engaged Users (M\SD)	Heavy Addicts (M\SD)
BSMAS (Total)	13.309/4.846	16.831/5.138	17.564/5.154	17.607/4.799
Depression	4.930/4.582	7.728/5.545	7.778/5.224	8.444/5.278
Anxiety	3.894/4.160	6.786/5.033	7.201/4.767	7.621/5.062
Stress	5.682/4.742	8.385/5.178	8.666/4.696	8.719/4.818
FoMO	18.534/6.330	22.424/8.038	23.260/7.262	24.321/7.487
Self-esteem	33.368/6.617	30.762/6.078	30.531/6.281	29.790/5.344

## Discussion

The primary objective of this study is to define latent subgroups or individual classes related to PSMU based on responses to the BSMAS items. The study also examined several psychological constructs that may be linked to PSMU latent class memberships and that have not been examined together before. In this context, anxiety, depression, stress, FOMO, and self-esteem have been analyzed in terms of their relationships with the latent profiles of PSMU.

**Hypothesis 1,** proposed that latent profiles in the range of three to five would emerge based on responses to the BSMAS items. In our study, four classes were identified, supporting H1. This finding aligns with previous studies emphasizing the heterogeneity of PSMU among adults [25, 61, 78, 82]. This result supports the four-class study by Cui et al., [78] conducted with Chinese university students aged 17–26. However, it differs from the three-class models found by Cheng et al., [25] in adults from English and American cultures with an average age of 43.62, Keum et al., [82] in American young adults aged 17–25, Bányai et al., [61] in Hungarian adolescents from 9th and 10th grades, and Ilakkuvan et al., [83] in young adults aged 18–24, who found five classes. The differences in these results are thought to be explained by the varying levels of needs in the context of SDT and individual differences in fulfilling these needs, the interaction of personality, emotion, cognition, and executive processes in the I-PACE theory, which differs among individuals, and the variability of psychopathology levels emphasized in the CIUT theory. In addition, it is thought that class differences are seen as a result of the effects of different cultural and contextual variables on needs and their meeting methods, as well as on structures such as personality.

**Hypothesis 2** proposed that the levels of stress, anxiety, depression, and FOMO would be positively correlated with the most severe latent profile memberships based on PSMU. The results we obtained show that stress, depression, and FOMO have positive and significant relationships with the most severe PSMU latent profile, Heavy Addicts/Profile 4. However, It was found that anxiety did not show a significant associated with either Profile 4 (the most severe PSMU profile) or any of the other profiles. When evaluating the hypothesis and results, it appears consistent with SDT, I-PACE, and CIUT that individuals engage with social media at problematic levels as a result of unfulfilled needs, the interaction of individual and cognitive-emotional characteristics, and the experience of psychopathology and FOMO. These results align with previous research findings, which suggest that depression [24–26], stress [31, 78, 92], and FOMO [40, 42] are associated with PSMU.

With respect to anxiety, the findings of the present study indicate a more nuanced pattern than previously suggested. Although anxiety was significantly and positively associated with PSMU at the correlational level, the person-centered analyses revealed that anxiety was not uniformly related to all latent profiles. Specifically, anxiety emerged as a significant positive correlate only for membership in the most severe PSMU profile (Heavy Addicts), while it did not significantly distinguish less severe profiles. This finding suggests that anxiety may play a particularly important role in more extreme forms of problematic social media engagement rather than in milder or moderate patterns of use. In line with this interpretation, prior research has documented that anxiety is more strongly linked to compulsive, uncontrolled, and avoidance-driven patterns of social media use, especially when social media functions as a maladaptive coping strategy for managing heightened emotional distress [28, 30, 93]. Furthermore, recent person-centered and severity-based studies have suggested that anxiety may become salient primarily at higher levels of problematic engagement, where impaired control, withdrawal, and conflict are more pronounced [78, 94]. From a theoretical perspective, this pattern is consistent with the I-PACE model, which posits that affective states such as anxiety may interact with maladaptive coping mechanisms and reinforcement processes, thereby facilitating the escalation from controlled or habitual use to more severe and compulsive forms of problematic use [95]. The present findings extend prior variable-centered research by demonstrating that the association between anxiety and PSMU is not uniform across individuals, but instead appears to be profile-specific, emerging most clearly among individuals characterized by the highest severity of problematic social media use.

**Hypothesis 3** suggested that self-esteem is anticipated to be negatively correlated with membership in the most severe latent profiles based on PSMU. The results we obtained showed that self-esteem had a negative and significant relationship with the most severe PSMU-based latent profile, Heavy Addicts/Profile 4, confirming Hypothesis 3. Previous studies also provide evidence supporting that people with low self-esteem are more likely to demonstrate higher levels of PSMU ([55, 58, 96]; Sommantico et al., 2023; [85]). Therefore, it can be said that PSMU is associated with a negative self-concept. This result is consistent with the overuse of social media to satisfy the need for competence as defined in SDT [97]. Individuals with low self-esteem support their self-worth through expressions of approval and affirmative sentiments from social media, while also fulfilling their competence needs. On the other hand, this finding is consistent with the I-PACE model, which posits that low self-esteem, considered a trait of personality, influences other cognitive-affective-executive factors such as distress, and FOMO, facilitating the emergence of PSMU [75]. When considered in the context of CIUT, It can be argued that those with low self-esteem are more prone to excessive social media use to compensate for it, which is aligns with the CIUT model [85].

In the study, we found that sex did not exhibit a notable association with any PSMU-based latent class memberships, while the age variable was negatively related to all PSMU-based class memberships. As participants’ ages increased, the possibility of belonging to a minimal user profile in terms of PSMU also increased. There is a lack of consensus in the existing literature concerning the relationship between age, sex, and PSMU. Some studies suggest that women ([98]; Monacıs et al., 2017; [99]) and younger individuals [100–102] are more prone to PSMU, while others argue that older adults [103] and men [104] are more susceptible. In the study by Stănculescu and Griffiths [105], it was concluded that age and sex variables have significant relationships with PSMU-based latent profile memberships. Some other studies also indicate that There is no meaningful association between PSMU and age or sex variables [106–108]. The results of our study seem to be consistent with researches suggesting that younger individuals, as part of the digital age, use technology-based communication tools more to enhance seek romantic relationships [109] and their social capital [110]. The findings regarding age and sex should be interpreted cautiously and in light of the mixed evidence reported in the literature. In the present study, age was negatively associated with membership in more problematic PSMU profiles, suggesting that younger adults were more likely to belong to higher-risk profiles. This finding is consistent with studies indicating that younger individuals tend to engage more intensively with digital technologies and social media platforms, potentially increasing vulnerability to problematic patterns of use. However, given the inconsistent findings across prior studies, this association should not be interpreted as a universal developmental trend. With respect to sex, no significant differences were observed across PSMU profile memberships. This result aligns with some previous research reporting minimal or non-significant sex effects, while contrasting with studies suggesting higher risk among either females or males. Importantly, the absence of sex differences in the present study should be interpreted with caution due to the sex imbalance in the sample, in which approximately two-thirds of participants were female. This imbalance may have limited the statistical power to detect sex-based differences and restricts the generalizability of the findings. Overall, these results underscore the importance of interpreting age and sex effects within specific sample characteristics and cultural contexts rather than drawing broad generalizations.

The present study makes several novel contributions to the growing person-centered literature on problematic social media use (PSMU). First, whereas previous latent profile and latent class analyses have predominantly focused on adolescents or university student samples (e.g., Bányai et al., [61], Marino, [63], the current study extends this line of research by identifying heterogeneous PSMU profiles across a wide adult age range within a large and demographically diverse sample. This finding suggests that distinct patterns of problematic social media use are not confined to developmental periods characterized by heightened social media engagement but may persist across adulthood, thereby extending the generalizability of earlier person-centered findings [18, 62]. Second, in contrast to prior person-centered studies that have typically examined a limited number of psychological correlates in isolation—most often focusing on anxiety, depression, or impulsivity [39, 63]—the present study adopts an integrative approach by simultaneously examining psychological distress, fear of missing out (FoMO), and self-esteem within a single analytical framework. This approach allows for a more nuanced understanding of how multiple psychological vulnerabilities co-occur within specific PSMU profiles. The finding that more problematic profiles are characterized by elevated distress and FoMO alongside lower self-esteem extends prior variable-centered and person-centered research by highlighting the cumulative psychological burden associated with severe patterns of PSMU [22, 59]. By situating the analysis within a Turkish cultural context, the present study contributes to the relatively limited body of non-Western person-centered research on problematic social media use. While the overall profile structure shows partial convergence with prior LPA studies conducted in Western contexts (e.g., Bányai et al., [61], Marino, [63], the specific psychological patterns associated with each profile underscore the importance of cultural and contextual factors in shaping problematic social media engagement. In this respect, the findings both replicate and extend existing person-centered models of PSMU by demonstrating that heterogeneity in problematic use manifests across different cultural and demographic contexts [20, 95].

### Implications

The identification of distinct latent profiles of problematic social media use (PSMU) has important implications for clinical practice, educational settings, and public health policy. In particular, the differentiation between profiles such as “Habitual Users” and “Heavy Addicts” provides a clinically meaningful framework for understanding varying levels of risk and psychological vulnerability. Individuals classified within the “Heavy Addicts” profile exhibited elevated levels of psychological distress (depression and stress), higher fear of missing out (FoMO), and lower self-esteem, suggesting a profile that may require more intensive psychological attention compared to less severe profiles.

From a clinical perspective, these findings suggest that mental health professionals may benefit from adopting a profile-informed screening approach to distinguish between different patterns of PSMU. The combined use of brief measures assessing PSMU severity (e.g., BSMAS), psychological distress, FoMO, and self-esteem may help clinicians differentiate individuals whose social media use is habitual but relatively controlled from those whose use is more severe and psychologically impairing. Such differentiation may be particularly useful in prioritizing assessment and intervention efforts. Moreover, intervention strategies may be tailored according to the dominant psychological characteristics associated with each profile. For example, PSMU patterns primarily associated with elevated stress and FoMO may benefit from cognitive-behavioral interventions targeting maladaptive social comparison, reassurance-seeking, and emotion regulation, whereas PSMU linked to low self-esteem may respond more effectively to interventions focusing on enhancing self-worth, perceived competence, and offline interpersonal functioning.

In educational contexts, the findings underscore the value of preventive and psychoeducational initiatives aimed at promoting balanced social media use, emotional regulation skills, and awareness of FoMO-related behaviors. Educators and school-based mental health professionals may use profile-based insights to identify students who exhibit early risk indicators of problematic social media use and to implement targeted digital well-being programs that support healthier patterns of online engagement.

At the public health and policy level, the identified PSMU profiles may inform the development of digital well-being and mental health promotion strategies in Türkiye. Policymakers and institutions may consider integrating profile-based risk indicators into national digital literacy programs, public awareness campaigns, and community-based mental health initiatives, particularly those targeting young adults and other vulnerable groups. Such profile-informed approaches may enhance the effectiveness of prevention efforts by acknowledging heterogeneity in problematic social media use rather than relying on one-size-fits-all interventions.

### Limitations and Recommendations for Future Research

This study contributes to the existing researches by identifying heterogeneity in PSMU. The current study provides a valuable contribution to the existing body of research on the subject by identifying heterogeneity in PSMU across individuals through the use of LPA.

Additionally, the study is important in revealing the relationship between variables like, stress, depression, anxiety, FOMO, and self-esteem, which are considered factors associated with PSMU, and latent class memberships. In this respect, the study differs from variable-centered correlational studies on PSMU that ignore heterogeneity across individuals. Moreover, unlike most other person-centered studies examining PSMU [61, 78, 82], this study does not have age-related limitations. The study encompasses a broad age range, with participants’ ages ranging from 17 to 68 years [36, 111–118].

In addition to the strengths mentioned above, this research has some limitations. First, almost two-thirds of the participants were women, which restricts the generalizability of the findings across different demographics. Future research is advised to incorporate study samples with greater representational power. The second limitation pertains to the employment of self-report scales to evaluate the variables in this study. Responses to these scales are based on subjective experiences rather than objective ones, potentially being influenced by social desirability bias and recall bias, which could lead to data distortion. In addition, because all variables were assessed using self-report measures, the observed associations may be partially influenced by common method variance, which can inflate relationships among constructs measured using the same method. Future studies could overcome this limitation by employing more objective diagnostic measures, such as structured clinical interviews based on DSM-5 criteria. Another limitation concerns the omission of other potentially relevant variables associated with PSMU. Factors such as personality traits (e.g., impulsivity, neuroticism), perceived social support, and comorbid psychiatric conditions were not assessed in the present study and may have contributed to differences in profile membership. Including these variables in future research may provide a more comprehensive understanding of the psychological and contextual processes underlying distinct PSMU profiles. In addition, the cultural context of the study should be considered when interpreting the findings. As the sample consisted solely of individuals from Türkiye, cultural norms, patterns of social media use, and contextual factors specific to this setting may limit the generalizability of the results to other populations. Replication studies across different cultural contexts are therefore needed to examine the cross-cultural robustness of the identified profiles.

One important limitation of the present study is its cross-sectional design, which restricts causal interpretation of the findings. Although higher levels of depression, stress, fear of missing out (FoMO), and lower self-esteem were associated with membership in more problematic PSMU profiles, these relationships cannot be assumed to be unidirectional. Prior research suggests that problematic social media use may both contribute to psychological distress and emerge as a coping response to existing emotional difficulties, indicating the potential for reciprocal or bidirectional relationships [2, 39]. Longitudinal and experimental studies are therefore needed to clarify the temporal ordering and causal dynamics underlying these associations. Future research employing longitudinal designs could examine how individuals transition between PSMU profiles over time, while experimental or intervention-based studies may help clarify the causal mechanisms linking psychological distress, FoMO, and self-esteem with problematic social media use.

## Conclusion

The present study demonstrates that problematic social media use (PSMU) is characterized by meaningful heterogeneity among individuals. Using a person-centered latent profile analysis approach, four distinct PSMU profiles were identified in a Turkish adult sample: Minimal Users, Habitual Users, Engaged Users, and Heavy Addicts. These profiles highlight qualitatively different patterns of social media engagement rather than variations along a single continuum.

The findings further indicate that higher levels of depression, stress, and fear of missing out (FoMO), along with lower self-esteem, were associated with a higher likelihood of membership in more problematic PSMU profiles, particularly the Heavy Addicts profile. In addition, anxiety emerged as a significant correlate specifically for the most severe PSMU profile, suggesting that anxiety may be more closely linked to extreme and compulsive patterns of problematic social media use rather than to milder forms of engagement. The findings further indicate that younger age was systematically associated with a higher likelihood of membership in more problematic PSMU profiles, suggesting that vulnerability to problematic patterns of social media use may be more pronounced in earlier adulthood. Accordingly, age, depression, stress, FoMO, self-esteem and anxiety should be understood as key psychological correlates of different PSMU styles rather than as causal determinants.

From an applied perspective, the identification of distinct PSMU profiles provides a useful framework for clinical, educational, and public health contexts. Profile-informed screening of psychological distress, FoMO, and self-esteem may assist clinicians, educators, and policymakers in identifying individuals at higher risk for problematic social media use and in tailoring prevention and intervention efforts to promote healthier patterns of social media engagement.

## Data Availability

The datasets used and/or analyzed during the current study available from the corresponding author on reasonable request.

## References

[1] Kaplan AM, Haenlein M. Users of the world, unite! The challenges and opportunities of social media. Bus Horiz. 2010;53(1):59–68. 10.1016/j.bushor.2009.09.003.

[2] Kuss DJ, Griffiths MD. Social Networking Sites and addiction: ten lessons learned. Int J Environ Res Public Health. 2017;14(3):311. 10.3390/ijerph14030311. 28304359 10.3390/ijerph14030311 PMC5369147

[3] DataReportal. 2024a. Retrieved on 02.11.2024 from https://datareportal.com/reports/digital-2024-deep-dive-5-billion-social-media-users

[4] DataReportal. 2024b. Retrieved on 02.11.2024 from https://datareportal.com/reports/digital-2024-turkey

[5] Ellison NB, Steinfield C, Lampe C. The benefits of Facebook friends: social capital and college students’ use of online social network sites. J Comput-Mediat Commun. 2007;12(4):1143–68. 10.1111/j.1083-6101.2007.00367.x.

[6] Verduyn P, Ybarra O, Résibois M, Jonides J, Kross E. Do social network sites enhance or undermine subjective well-being? A critical review. Soc Issues Policy Rev. 2017;11(1):274–302. 10.1111/sipr.12033.

[7] Valkenburg PM, Peter J. Online communication among adolescents: an integrated model of its attraction, opportunities, and risks. J Adolesc Health. 2011;48(2):121–7. 10.1016/j.jadohealth.2010.08.020. 21257109 10.1016/j.jadohealth.2010.08.020

[8] Griffiths MD. Social networking addiction: Emerging themes and issues. J Addict Res Ther. 2013;4(5). 10.4172/2155-6105.1000e118.

[9] Griffiths M. A ‘components’ model of addiction within a biopsychosocial framework. J Subst Use. 2005;10(4):191–7. 10.1080/14659890500114359.

[10] Luo T, Qin L, Cheng L, Wang S, Zhu Z, Xu J, et al. Determination the cut-off point for the Bergen social media addiction (BSMAS): Diagnostic contribution of the six criteria of the components model of addiction for social media disorder. J Behav Addict. 2021;10(2):281–90. 10.1556/2006.2021.00025. 34010148 10.1556/2006.2021.00025 PMC8996805

[11] Sumen A, Evgin D. Social media addiction in high school students: a cross-sectional study examining its relationship with sleep quality and psychological problems. Child Indic Res. 2021;14(6):2265–83. 10.1007/s12187-021-09838-9. 34367373 10.1007/s12187-021-09838-9 PMC8329411

[12] Steers MLN, Wickham RE, Acitelli LK. Seeing everyone else’s highlight reels: how Facebook usage is linked to depressive symptoms. J Soc Clin Psychol. 2014;33(8):701–31. 10.1521/jscp.2014.33.8.701.

[13] Lee DS, Jiang T, Crocker J, Way BM. Social media use and its link to physical health indicators. Cyberpsychol Behav Soc Netw. 2022;25(2):87–93. 10.1089/cyber.2021.0188. 35021894 10.1089/cyber.2021.0188 PMC8864418

[14] Monacis L, Griffiths MD, Limone P, Sinatra M, Servidio R. Selfitis behavior: assessing the Italian version of the Selfitis Behavior Scale and its mediating role in the relationship of dark traits with social media addiction. Int J Environ Res Public Health. 2020;17(16):5738. 10.3390/ijerph17165738. 32784419 10.3390/ijerph17165738 PMC7460134

[15] Moreno MA, Gannon K. Social media and health. Adolesc Med State Art Rev. 2013;24(3):538–52. 10.1542/9781581108736-social_media. 24654547

[16] Marttila E, Koivula A, Räsänen P. Does excessive social media use decrease subjective well-being? A longitudinal analysis of the relationship between problematic use, loneliness and life satisfaction. Telemat Informatics. 2021;59:101556. 10.1016/j.tele.2020.101556.

[17] Ho TTQ. Facebook addiction and depression: loneliness as a moderator and poor sleep quality as a mediator. Telemat Informatics. 2021;61:101617. 10.1016/j.tele.2021.101617.

[18] Andreassen CS, Pallesen S, Griffiths MD. The relationship between addictive use of social media, narcissism, and self-esteem: findings from a large national survey. Addict Behav. 2017;64:287–93. 10.1016/j.addbeh.2016.03.006. 27072491 10.1016/j.addbeh.2016.03.006

[19] Andreassen CS, Pallesen S. Social network site addiction-an overview. Curr Pharm Des. 2014;20(25):4053–61. 10.2174/13816128113199990616. 24001298 10.2174/13816128113199990616

[20] Kardefelt-Winther D, Heeren A, Schimmenti A, Van Rooij A, Maurage P, Carras M, et al. How can we conceptualize behavioural addiction without pathologizing common behaviours? Addiction. 2017;112(10):1709–15. 10.1111/add.13763. 28198052 10.1111/add.13763 PMC5557689

[21] Billieux J, Maurage P, Lopez-Fernandez O, Kuss DJ, Griffiths MD. Can disordered mobile phone use be considered a behavioral addiction? An update on current evidence and a comprehensive model for future research. Curr Addict Rep. 2015;2(2):156–62. 10.1007/s40429-015-0054-y.

[22] Andreassen CS, Billieux J, Griffiths MD, Kuss DJ, Demetrovics Z, Mazzoni E, Pallesen S. The relationship between addictive use of social media and video games and symptoms of psychiatric disorders: A large-scale cross-sectional study. Psychol Addict Behav. 2016;30(2):252–62. 10.1037/adb0000160. 26999354 10.1037/adb0000160

[23] Drapeau A, Marchand A, Beaulieu-Prévost D. Epidemiology of psychological distress. Mental İllnesses-Understanding Prediction Control. 2012;69(2):105–6. 10.5772/30872.

[24] Bettmann JE, Anstadt G, Casselman B, Ganesh K. Young adult depression and anxiety linked to social media use: assessment and treatment. Clin Soc Work J. 2021;49:368–79. 10.1007/s10615-020-00752-1.

[25] Cheng C, Ebrahimi OV, Luk JW. Heterogeneity of prevalence of social media addiction across multiple classification schemes: latent profile analysis. J Med Internet Res. 2022;24(1):e27000. 10.2196/27000. 35006084 10.2196/27000 PMC8787656

[26] Haand R, Shuwang Z. The relationship between social media addiction and depression: a quantitative study among university students in Khost, Afghanistan. Int J Adolesc Youth. 2020;25(1):780–6. 10.1080/02673843.2020.1741407.

[27] McCrae N, Gettings S, Purssell E. Social media and depressive symptoms in childhood and adolescence: a systematic review. Adolesc Res Rev. 2017;2:315–30. 10.1007/s40894-017-0053-4.

[28] Hussain Z, Griffiths MD. Problematic social networking site use and comorbid psychiatric disorders: a systematic review of recent large-scale studies. Front Psychiatry. 2018;9:686. 10.3389/fpsyt.2018.00686. 30618866 10.3389/fpsyt.2018.00686 PMC6302102

[29] Lopes LS, Valentini JP, Monteiro TH, Costacurta MCDF, Soares LON, Telfar-Barnard L, et al. Problematic social media use and its relationship with depression or anxiety: a systematic review. Cyberpsychol Behav Soc Netw. 2022;25(11):691–702. 10.1089/cyber.2021.0300. 36219756 10.1089/cyber.2021.0300

[30] Vannucci A, Flannery KM, Ohannessian CM. Social media use and anxiety in emerging adults. J Affect Disord. 2017;207:163–6. 10.1016/j.jad.2016.08.040. 27723539 10.1016/j.jad.2016.08.040

[31] Hussain Z, Griffiths MD. The associations between problematic social networking site use and sleep quality, attention-deficit hyperactivity disorder, depression, anxiety and stress. Int J Ment Health Addict. 2021;19(3):686–700. 10.1007/s11469-019-00175-1.

[32] Keles B, McCrae N, Grealish A. A systematic review: the influence of social media on depression, anxiety and psychological distress in adolescents. Int J Adolesc Youth. 2020;25(1):79–93. 10.1080/02673843.2019.1590851.

[33] Primack BA, Shensa A, Sidani JE, Whaite EO, yi, Lin L, Rosen D, Miller E. (2017). Social media use and perceived social isolation among young adults in the US. *American Journal of Preventive Medicine*, 53(1), 1–8. 10.1016/j.amepre.2017.01.010 10.1016/j.amepre.2017.01.010 PMC5722463 28279545

[34] Lin LY, Sidani JE, Shensa A, Radovic A, Miller E, Colditz JB, et al. Associatıon between social media use and depression among U.S. young adults. Depress Anxiety. 2016;33(4):323–31. 10.1002/da.22466. 26783723 10.1002/da.22466 PMC4853817

[35] Keles E. Use of Facebook for the Community Services Practices course: community of inquiry as a theoretical framework. Comput Educ. 2018;116:203–24. 10.1016/j.compedu.2017.09.003.

[36] Przybylski AK, Murayama K, DeHaan CR, Gladwell V. Motivational, emotional, and behavioral correlates of fear of missing out. Comput Human Behav. 2013;29(4):1841–8. 10.1016/j.chb.2013.02.014.

[37] Yuan G, Elhai JD, Hall BJ. The influence of depressive symptoms and fear of missing out on severity of problematic smartphone use and Internet gaming disorder among Chinese young adults: a three-wave mediation model. Addict Behav. 2021;112:106648. 10.1016/j.addbeh.2020.106648. 32977268 10.1016/j.addbeh.2020.106648

[38] Elhai JD, Levine JC, Dvorak RD, Hall BJ. Fear of missing out, need for touch, anxiety and depression are related to problematic smartphone use. Comput Hum Behav. 2016;63:509–16. 10.1016/j.chb.2016.05.079.

[39] Elhai JD, Yang H, Fang J, Bai X, Hall BJ. Depression and anxiety symptoms are related to problematic smartphone use severity in Chinese young adults: fear of missing out as a mediator. Addict Behav. 2020;101:105962. 10.1016/j.addbeh.2019.04.020. 31030950 10.1016/j.addbeh.2019.04.020

[40] Fabris MA, Marengo D, Longobardi C, Settanni M. Investigating the links between fear of missing out, social media addiction, and emotional symptoms in adolescence: the role of stress associated with neglect and negative reactions on social media. Addict Behav. 2020;106:106364. 10.1016/j.addbeh.2020.106364. 32145495 10.1016/j.addbeh.2020.106364

[41] Fang J, Wang X, Wen Z, Zhou J. Fear of missing out and problematic social media use as mediators between emotional support from social media and phubbing behavior. Addict Behav. 2020;107:106430. 10.1016/j.addbeh.2020.106430. 32289745 10.1016/j.addbeh.2020.106430

[42] Topino E, Gori A, Jimeno MV, Ortega B, Cacioppo M. The relationship between social media addiction, fear of missing out and family functioning: a structural equation mediation model. BMC Psychol. 2023;11(1):383. 10.1186/s40359-023-01409-7. 37941011 10.1186/s40359-023-01409-7 PMC10634114

[43] Sultan AJ. Fear of missing out and self-disclosure on social media: the paradox of tie strength and social media addiction among young users. Young Consum. 2021;22(4):555–77. 10.1108/YC-10-2020-1233.

[44] Tandon A, Dhir A, Talwar S, Kaur P, Mäntymäki M. Dark consequences of social media-induced fear of missing out (FoMO): social media stalking, comparisons, and fatigue. Technol Forecast Soc Change. 2021;171:120931. 10.1016/j.techfore.2021.120931.

[45] Varchetta M, Fraschetti A, Mari E, Giannini AM. Social media addiction, fear of missing out (FoMO) and online vulnerability in university students. Revista Digital de Investigación en Docencia Universitaria. 2020;14(1):e1187. 10.19083/ridu.2020.1187.

[46] Fioravanti G, Casale S, Benucci SB, Prostamo A, Falone A, Ricca V, et al. Fear of missing out and social networking sites use and abuse: a meta-analysis. Computers Hum Behav. 2021;122:106839. 10.1016/j.chb.2021.106839.

[47] Adams SK, Murdock KK, Daly-Cano M, Rose M. Sleep in the social world of college students: bridging interpersonal stress and fear of missing out with mental health. Behav Sci. 2020;10(2):54. 10.3390/bs10020054. 32041120 10.3390/bs10020054 PMC7071423

[48] Wolniewicz CA, Rozgonjuk D, Elhai JD. Boredom proneness and fear of missing out mediate relations between depression and anxiety with problematic smartphone use. Hum Behav Emerg Technol. 2020;2(1):61–70. 10.1002/hbe2.159.

[49] Rozgonjuk D, et al. Individual differences in fear of missing out (FoMO): age, gender, and the Big Five personality trait domains, facets, and items. Pers Individ Differ. 2021;171:110546. 10.1016/j.paid.2020.110546.

[50] Oberst U, Wegmann E, Stodt B, Brand M, Chamarro A. Negative consequences from heavy social networking in adolescents: the mediating role of fear of missing out. J Adolesc. 2017;55:51–60. 10.1016/j.adolescence.2016.12.008. 28033503 10.1016/j.adolescence.2016.12.008

[51] Minev M, Petrova B, Mineva K, Petkova M, Strebkova R. Self-esteem in adolescents. Trakia J Sci. 2018;16(2):114–8. 10.15547/tjs.2018.02.008.

[52] Rosenberg M. Society and the adolescent self-image. Princeton University Press; 1965. 10.1515/9781400876136.

[53] Kircaburun K. Self-esteem, daily ınternet use and social media addiction as predictors of depression among turkish adolescents. J Educ Pract. 2016;7(24):64–72.

[54] Orth U, Robins RW, Roberts BW. Low self-esteem prospectively predicts depression in adolescence and young adulthood. J Pers Soc Psychol. 2008;95(3):695. 10.1037/0022-3514.95.3.695. 18729703 10.1037/0022-3514.95.3.695

[55] Acar IH, Avcılar G, Yazıcı G, Bostancı S. The roles of adolescents’ emotional problems and social media addiction on their self-esteem. Curr Psychol. 2022;41(10):6838–47. 10.1007/s12144-020-01174-5.

[56] Baturay MH, Toker S. Self-esteem shapes the impact of GPA and general health on Facebook addiction: a mediation analysis. Soc Sci Comput Rev. 2017;35(5):555–75. 10.1177/0894439316656606.

[57] Błachnio A, Przepiorka A. Be aware! If you start using Facebook problematically you will feel lonely: phubbing, loneliness, self-esteem, and Facebook intrusion. A cross-sectional study. Soc Sci Comput Rev. 2019;37(2):270–8. 10.1177/0894439318754490.

[58] Colak M, Bingol OS, Dayi A. Self-esteem and social media addiction level in adolescents: the mediating role of body image. Indian J Psychiatry. 2023;65(5):595–600. 10.4103/indianjpsychiatry.indianjpsychiatry_306_22. 37397839 10.4103/indianjpsychiatry.indianjpsychiatry_306_22 PMC10309264

[59] Błachnio A, Przepiorka A, Pantic I. Association between Facebook addiction, self-esteem and life satisfaction: a cross-sectional study. Comput Human Behav. 2016;55:701–5. 10.1016/j.chb.2015.10.026.

[60] Bergagna E, Tartaglia S. Self-esteem, social comparison, and Facebook use. Europe’s J Psychol. 2018;14(4):831. 10.5964/ejop.v14i4.1592. 10.5964/ejop.v14i4.1592 PMC6266525 30555588

[61] Bányai F, Zsila Á, Király O, Maraz A, Elekes Z, Griffiths MD, et al. Problematic social media use: results from a large-scale nationally representative adolescent sample. PLoS One. 2017;12(1):e0169839. 10.1371/journal.pone.0169839. 28068404 10.1371/journal.pone.0169839 PMC5222338

[62] Dhir A, Yossatorn Y, Kaur P, Chen S. Online social media fatigue and psychological wellbeing-a study of compulsive use, fear of missing out, fatigue, anxiety and depression. Int J Inf Manag. 2018;40:141–52. 10.1016/j.ijinfomgt.2018.01.012.

[63] Marino C, Gini G, Vieno A, Spada MM. A comprehensive meta-analysis on problematic Facebook use. Comput Hum Behav. 2018;83:262–77. 10.1016/j.chb.2018.02.009.

[64] Lee Y, Yoo S. Individual profiles and team classes of the climate for creativity: a multilevel latent profile analysis. Creat Innov Manag. 2020;29(3):438–52. 10.1111/caim.12371.

[65] Jung T, Wickrama KA. An introduction to latent class growth analysis and growth mixture modeling. Soc Pers Psychol Compass. 2008;2(1):302–17. 10.1111/j.1751-9004.2007.00054.x.

[66] Ferguson SL, Moore G, E. W., Hull DM. Finding latent groups in observed data: A primer on latent profile analysis in Mplus for applied researchers. Int Journal Behav Development. 2020;44(5):458–68. 10.1177/0165025419881721.

[67] Deci EL, Ryan RM. The general causality orientations scale: self-determination in personality. J Res Pers. 1985;19(2):109–34. 10.1016/0092-6566(85)90023-6.

[68] Sun Y, Zhang Y. A review of theories and models applied in studies of social media addiction and implications for future research. Addict Behav. 2021;114:106699. 10.1016/j.addbeh.2020.106699. 33268185 10.1016/j.addbeh.2020.106699

[69] Zare L, Firouzi M, Taghvaeinia A, Zadehbagheri G. The relationship between basic psychological needs and internet addiction with the moderating role of problem-oriented coping style. Int J Behav Sci. 2021;15(3):162–7. 10.30491/ijbs.2021.269410.1464.

[70] Soenens B, Vansteenkiste M. (2011). When is identity congruent with the self? A self-determination theory perspective. In *Handbook of identity theory and research* (pp. 381–402). Springer New York. 10.1007/978-1-4419-7988-9_17

[71] O’Farrell DL, Baynes KL, Pontes M, Griffiths HD, M., Stavropoulos V. Depression and disordered gaming: does culture matter? Int J Mental Health Addict. 2020;1–19. 10.1007/s11469-020-00231-1.

[72] Yen JY, Lin HC, Chou WP, Liu TL, Ko CH. Associations among resilience, stress, depression, and internet gaming disorder in young adults. Int J Environ Res Public Health. 2019;16(17):3181. 10.3390/ijerph16173181. 31480445 10.3390/ijerph16173181 PMC6747224

[73] Marengo D, Montag C, Sindermann C, Elhai JD, Settanni M. Examining the links between active Facebook use, received likes, self-esteem and happiness: a study using objective social media data. Telemat Informatics. 2021;58:101523. 10.1016/j.tele.2020.101523.

[74] Brand M, Young KS, Laier C, Wölfling K, Potenza MN. Integrating psychological and neurobiological considerations regarding the development and maintenance of specific Internet-use disorders: an interaction of person-affect-cognition-execution (I-PACE) model. Neurosci Biobehav Rev. 2016;71:252–66. 10.1016/j.neubiorev.2016.08.033. 27590829 10.1016/j.neubiorev.2016.08.033

[75] Seabrook EM, Kern ML, Rickard NS. Social networking sites, depression, and anxiety: a systematic review. JMIR Ment Health. 2016;3(4):e5842. 10.2196/mental.5842. 10.2196/mental.5842 PMC5143470 27881357

[76] Elhai JD, Hall BJ, Erwin MC. Emotion regulation’s relationships with depression, anxiety and stress due to imagined smartphone and social media loss. Psychiatr Res. 2018;261:28–34. 10.1016/j.psychres.2017.12.045. 10.1016/j.psychres.2017.12.045 29276991

[77] Kardefelt-Winther D. The moderating role of psychosocial well-being on the relationship between escapism and excessive online gaming. Comput Hum Behav. 2014;38:68–74. 10.1016/j.chb.2014.05.020.

[78] Cui J, Wang Y, Liu D, Yang H. Depression and stress are associated with latent profiles of problematic social media use among college students. Front Psychiatry. 2023;14:1306152. 10.3389/fpsyt.2023.1306152. 38098636 10.3389/fpsyt.2023.1306152 PMC10720731

[79] Andreassen CS, Torsheim T, Brunborg GS, Pallesen S. Development of a Facebook addiction scale. Psychol Rep. 2012;110(2):501–17. 10.2466/02.09.18.PR0.110.2.501-517. 22662404 10.2466/02.09.18.PR0.110.2.501-517

[80] Müller SM, Wegmann E, Arias MG, Brotóns EB, Giráldez CM, Brand M. Deficits in executive functions but not in decision making under risk in individuals with problematic social-network use. Compr Psychiatry. 2021;106:152228. 10.1016/j.comppsych.2021.152228. 33581450 10.1016/j.comppsych.2021.152228

[81] Hong L, Lai X, Xu D, Zhang W, Wu B, Yu X, et al. Distinct patterns of problematic smartphone use and related factors in Chinese college students. BMC Psychiatry. 2022;22(1):747. 10.1186/s12888-022-04395-z. 36451113 10.1186/s12888-022-04395-z PMC9710163

[82] Keum BT, Wang YW, Callaway J, Abebe I, Cruz T, O’Connor S. Benefits and harms of social media use: a latent profile analysis of emerging adults. Curr Psychol. 2023;42(27):23506–18. 10.1007/s12144-022-03473-5. 10.1007/s12144-022-03473-5 PMC9302950 35891891

[83] Ilakkuvan V, Johnson A, Villanti AC, Evans WD, Turner M. Patterns of social media use and their relationship to health risks among young adults. J Adolesc Health. 2019;64(2):158–64. 10.1016/j.jadohealth.2018.06.025. 30269907 10.1016/j.jadohealth.2018.06.025 PMC12833830

[84] Soraci P, Ferrari A, Barberis N, Luvarà G, Urso A, Del Fante E, et al. Psychometric analysis and validation of the Italian Bergen Facebook Addiction Scale. Int J Ment Health Addict. 2023;21(1):451–67. 10.1007/s11469-020-00346-5.

[85] Soraci P, Pisanti R, Servidio R, Holte AJ, Ferrari A, Demetrovics Z, et al. The associations between the problematic social media and smartphone use, social phobia, and self-esteem: a structural equation modeling analysis. Int J Ment Health Addict. 2024. 10.1007/s11469-024-01375-0.

[86] Demirci I. The adaptation of the Bergen Social Media Addiction Scale to Turkish and its evaluation of relationship with depression and anxiety symptoms. Anatol J Psychiatry. 2019;20(1):15–22. 10.5455/apd.41585.

[87] Henry JD, Crawford JR. The short-form version of the depression anxiety stress scales (DASS-21): construct validity and normative data in a large non-clinical sample. Br J Clin Psychol. 2005;44(2):227–39. 10.1348/014466505X29657. 16004657 10.1348/014466505X29657

[88] Mahmoud JSR, Staten RT, Hall LA, Lennie TA. The relationship among young adult college students’ depression, anxiety, stress, demographics, life satisfaction, and coping styles. Issues Ment Health Nurs. 2012;33(3):149–56. 10.3109/01612840.2011.632708. 22364426 10.3109/01612840.2011.632708

[89] Yılmaz O, Boz H, Arslan A. The validity and reliability of depression stress and anxiety scale (DASS-21) Turkish short form. Res Financial Economic Social Stud. 2017;2(2):78–91.

[90] Can G, Satici SA. Adaptation of fear of missing out scale (FoMOs): Turkish version validity and reliability study. Psicologia: Reflexão e Cr’ıtica. 2019;32(3):1–7. 10.1186/s41155-019-0117-4. 10.1186/s41155-019-0117-4 PMC6967379 32026206

[91] Çuhadaroğlu Ö. (1986). *Self-esteem in adolescents.* Unpublished doctoral dissertation, Hacettepe University Faculty of Medicine, Department of Psychiatry, Ankara.

[92] Peng P, Liao Y. Six addiction components of problematic social media use in relation to depression, anxiety, and stress symptoms: a latent profile analysis and network analysis. BMC Psychiatry. 2023;23(1):321. 10.1186/s12888-023-04837-2. 37158854 10.1186/s12888-023-04837-2 PMC10166459

[93] Elhai JD, Yang H, Montag C. Cognitive-and emotion-related dysfunctional coping processes: transdiagnostic mechanisms explaining depression and anxiety’s relations with problematic smartphone use. Curr Addict Rep. 2019;6(4):410–7. 10.1007/s40429-019-00260-4.

[94] Pontes HM, Taylor M, Stavropoulos V. Beyond Facebook addiction: the role of cognitive-related factors and psychiatric distress in social networking site addiction. Cyberpsychol Behav Soc Netw. 2018;21(4):240–7. 10.1089/cyber.2017.0609. 29589972 10.1089/cyber.2017.0609

[95] Brand M, Wegmann E, Stark R, Müller A, Wölfling K, Robbins TW, et al. The interaction of person-affect-cognition-execution (I-PACE) model for addictive behaviors: update, generalization to addictive behaviors beyond internet-use disorders, and specification of the process character of addictive behaviors. Neurosci Biobehav Rev. 2019;104:1–10. 10.1016/j.neubiorev.2019.06.032. 31247240 10.1016/j.neubiorev.2019.06.032

[96] Uram P, Skalski S. Still logged in? The link between Facebook addiction, FoMO, self-esteem, life satisfaction and loneliness in social media users. Psychol Rep. 2022;125(1):218–31. 10.1177/0033294120980970. 33302798 10.1177/0033294120980970

[97] Dadiotis A, Roussos P. Relationship between FoMO, problematic social media use, self-esteem, negative affectivity, and physical exercise: a structural equation model. J Technol Behav Sci. 2024;9(2):313–24. 10.1007/s41347-023-00340-3.

[98] Kircaburun K, Griffiths MD. Instagram addiction and the Big Five of personality: the mediating role of self-liking. J Behav Addict. 2018;7(1):158–70. 10.1556/2006.7.2018.15. 29461086 10.1556/2006.7.2018.15 PMC6035031

[99] Su W, Han X, Yu H, Wu Y, Potenza MN. Do men become addicted to internet gaming and women to social media? A meta-analysis examining gender-related differences in specific internet addiction. Comput Hum Behav. 2020;113:106480. 10.1016/j.chb.2020.106480.

[100] Abbasi IS. Social media addiction in romantic relationships: does user’s age influence vulnerability to social media infidelity? Pers Individ Differ. 2019;139:277–80. 10.1016/j.paid.2018.10.038.

[101] Griffiths MD, Kuss DJ, Demetrovics Z. Social networking addiction: An overview of preliminary findings. Behav Addictions. 2014;119–41. 10.1016/B978-0-12-407724-9.00006-9.

[102] Shensa A, Escobar-Viera CG, Sidani JE, Bowman ND, Marshal MP, Primack BA. Problematic social media use and depressive symptoms among US young adults: a nationally-representative study. Soc Sci Med. 2017;182:150–7. 10.1016/j.socscimed.2017.03.061. 28446367 10.1016/j.socscimed.2017.03.061 PMC5476225

[103] Floros G, Siomos K. The relationship between optimal parenting, Internet addiction and motives for social networking in adolescence. Psychiatr Res. 2013;209(3):529–34. 10.1016/j.psychres.2013.01.010. 10.1016/j.psychres.2013.01.010 23415042

[104] Çam E, Isbulan O. A new addiction for teacher candidates: social networks. Turkish Online J Educational Technology-TOJET. 2012;11(3):14–9.

[105] Stănculescu E, Griffiths MD. Social media addiction profiles and their antecedents using latent profile analysis: the contribution of social anxiety, gender, and age. Telemat Informatics. 2022;74:101879. 10.1016/j.tele.2022.101879.

[106] Huang C. A meta-analysis of the problematic social media use and mental health. Int J Soc Psychiatry. 2022;68(1):12–33. 10.1177/0020764020978434. 33295241 10.1177/0020764020978434

[107] Sayili U, Pirdal BZ, Kara B, Acar N, Camcioglu E, Yilmaz E, Erginoz E. Internet addiction and social media addiction in medical faculty students: prevalence, related factors, and association with life satisfaction. J Community Health. 2023;48(2):189–98. 10.1007/s10900-022-01153-w. 36344767 10.1007/s10900-022-01153-w

[108] Tang J, Chang Y, Aggarwal C, Liu H. A survey of signed network mining in social media. ACM Comput Surveys (CSUR). 2016;49(3):1–37. 10.1145/2956185.

[109] Stănculescu E, Griffiths MD. Anxious attachment and Facebook addiction: the mediating role of need to belong, self-esteem, and Facebook use to meet romantic partners. Int J Ment Health Addict. 2021;(1):17. 10.1007/s11469-021-00598-9.

[110] Allen KA, Ryan T, Gray DL, McInerney DM, Waters L. Social media use and social connectedness in adolescents: the positives and the potential pitfalls. Educ Dev Psychol. 2014;31(1):18–31. 10.1017/edp.2014.2.

[111] Ali F, Ali A, Iqbal A, Zafar AU. How socially anxious people become compulsive social media users: the role of fear of negative evaluation and rejection. Telemat Inform. 2022;63:101658. 10.1016/j.tele.2021.101658.

[112] Billieux J, King DL, Higuchi S, Achab S, Bowden-Jones H, Hao W, et al. Functional impairment matters in the screening and diagnosis of gaming disorder: commentary on: scholars’ open debate paper on the World Health Organization ICD-11 Gaming Disorder proposal (Aarseth et al). J Behav Addict. 2017;6(3):285–9. 10.1556/2006.6.2017.036. 28816514 10.1556/2006.6.2017.036 PMC5700712

[113] George D, Mallery M. (2010). SPSS for windows step by step: A simple guide and reference, 17.0 update (10th ed.). Pearson.

[114] Koca F, Saatçı F. The mediator role of fear of missing out in the parent-adolescent relationship quality and problematic Internet use. Int J Ment Health Addict. 2022;20(3):1897–912. 10.1007/s11469-022-00822-0. 35465026 10.1007/s11469-022-00822-0 PMC9017719

[115] Monacis L, De Palo V, Griffiths MD, Sinatra M. Social networking addiction, attachment style, and validation of the Italian version of the Bergen Social Media Addiction Scale. J Behav Addict. 2017;6(2):178–86. 10.1556/2006.6.2017.023. 28494648 10.1556/2006.6.2017.023 PMC5520120

[116] Pang H, Pang H. (2022). Connecting mobile social media with psychosocial well-being: Understanding relationship between WeChat involvement, network characteristics, online capital and life satisfaction. *Social Networks*, *68*, 256–263. 10.1016/j.socnet.2021.08.006

[117] Rozgonjuk D, Levine JC, Hall BJ, Elhai JD. The association between problematic smartphone use, depression and anxiety symptom severity, and objectively measured smartphone use over one week. Comput Hum Behav. 2018;87:10–7. 10.1016/j.chb.2018.05.019.

[118] Sommantico M, Ramaglia F, Lacatena M. Relationships between depression, fear of missing out and social media addiction: the mediating role of self-esteem. Healthcare. 2023;11(12):1667. 10.3390/healthcare11121667. 37372785 10.3390/healthcare11121667 PMC10298336

